# The global epidemiology of hepatocellular carcinoma

**DOI:** 10.1097/HC9.0000000000000932

**Published:** 2026-04-13

**Authors:** Muhammad Ali Butt, Elizabeth S. Aby, Jose D. Debes

**Affiliations:** 1 Division of Gastroenterology, Hepatology and Nutrition, Transplant Hepatology, University of Minnesota, Minneapolis, Minnesota, USA; 2 Division of Gastroenterology, Department of Medicine, University of Minnesota, Minneapolis, Minnesota, USA; 3 Department of Gastroenterology, Erasmus MC, Rotterdam, The Netherlands

**Keywords:** age-standardized incidence rate, alcohol-associated liver disease, etiology, global epidemiology, hepatocellular carcinoma, metabolic dysfunction–associated steatotic liver disease, viral hepatitis

## Abstract

Hepatocellular carcinoma (HCC) remains a major global cause of cancer morbidity and mortality, with marked geographic heterogeneity in incidence and outcomes. The highest age-standardized incidence and mortality rates persist in East Asia and across much of Africa. In contrast, Europe and North America exhibit moderate incidence, while Latin America and Oceania represent intermediate-burden regions with important subregional heterogeneity, with higher mortality concentrated in underserved, rural, Indigenous, and remote populations. Globally, the etiologic landscape of HCC is shifting from predominantly infection-related toward metabolic dysfunction and alcohol-associated liver disease. Rural–urban disparities further exacerbate global HCC burden through gaps in vaccination coverage, antiviral access, diagnostic infrastructure, and specialty care, leading to later-stage presentation and poorer outcomes. In this review, we describe the epidemiological changes in HCC across different areas of the world, focusing on region-specific issues and identifying key aspects of epidemiological transition.

## Graphical abstract

**Figure:**
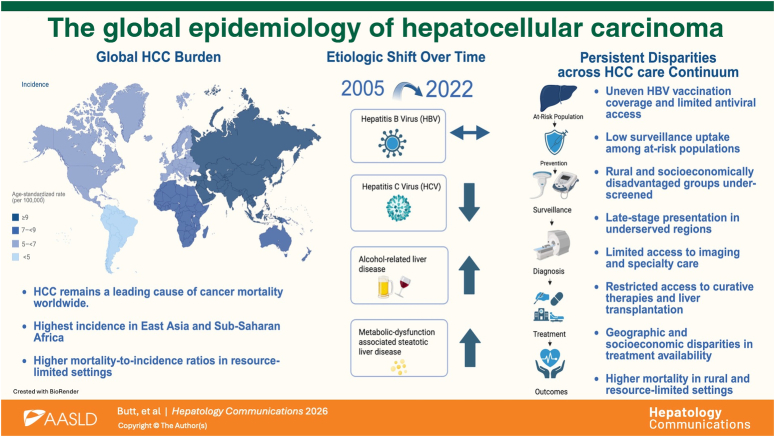

## INTRODUCTION

Liver cancer is the sixth most common malignancy and the third leading cause of cancer-related mortality worldwide.^1^ Hepatocellular carcinoma (HCC), the predominant histological subtype, accounts for over 80% of all primary liver cancers.^2^ According to GLOBOCAN 2022, there were an estimated 866,136 new liver cancer cases and 758,725 deaths globally, with a worldwide mortality-to-incidence ratio (MIR) of 0.86.^1^ Projections suggest that without intervention, the burden will double to 1.52 million new cases and 1.37 million deaths by 2050, underscoring HCC as a major global health challenge.^1^

**FIGURE 1:**
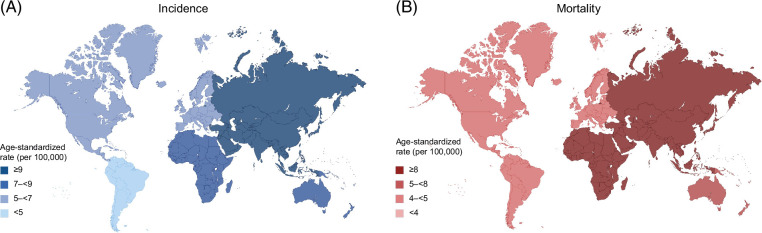
Global distribution of age-standardized incidence rates of hepatocellular carcinoma (HCC) in 2022. Rates are age-standardized to the world standard population. Substantial geographic heterogeneity is observed, with the highest incidence and mortality rates concentrated in Asia and parts of Africa, compared with other world regions. Data source: GLOBOCAN 2022, International Agency for Research on Cancer (Global Cancer Observatory). Abbreviations: ASIR, age-standardized incidence rates; ASMR, age-standardized mortality rates.

Marked geographic variation exists in the distribution of HCC. For instance, Eastern Asia (14.8–17.8 per 100,000), Northern Africa (13.2–15.2 per 100,000), and South-Eastern Asia (9.5–13.7 per 100,000) exhibit the highest Age-Standardized Incidence Rate (ASIR).^1^ In contrast, Europe and North America report moderate incidence rates (5–8 per 100,000), whereas Latin America and Oceania display lower global rates but with regional variability^1^ (Figure 1). This variation reflects differences in the prevalence and timing of exposure to key risk factors, including but not limited to viral hepatitis, metabolic dysfunction–associated steatotic liver disease (MASLD), alcohol-associated liver disease (ALD), and environmental factors, as well as disparities in healthcare resources, early detection capabilities, and access to potentially curative treatments.

This review synthesizes current global and regional trends in HCC, focusing on the evolving contributions of major risk factors, the impact of hepatitis B virus (HBV) vaccination, and hepatitis C virus (HCV) treatment programs, and the emerging importance of MASLD and ALD in shaping future global trends.

## SHIFTS IN ETIOLOGICAL DISTRIBUTION WORLDWIDE

The etiologic landscape of HCC is undergoing a profound transformation. Once predominantly attributed to chronic viral hepatitis, the burden is now increasingly driven by MASLD and ALD. Universal HBV vaccination, antiviral therapy for chronic HBV, curative treatment for HCV, early detection of MASLD, and alcohol-use reduction policies are proven strategies that collectively decrease the risk of CLD and subsequent HCC development.^3^^,^^4^ Despite these gains, HBV remains the leading cause of HCC globally, accounting for ~39% of all cases in 2022, with only a modest decline projected to 36.9% by 2050 due to challenges in vaccine implementation, particularly in rural areas.^4^ HCV-related HCC, comprising 29.1% of cases in 2022, is similarly expected to decrease to 25.9% by 2050 with expanded direct-acting antiviral (DAA) access.^4^ In contrast, rising rates of MASLD and harmful alcohol use are driving a growing proportion of HCC attributable to ALD and MASLD: alcohol-associated HCC is projected to increase from 18.8% to 21.1% of cases by 2050, while MASLD-related HCC is expected to rise from 8.0% to 10.8%,^4^ reflecting global epidemics of obesity, diabetes, and alcohol use, alongside persistent inequities in prevention, surveillance, and treatment across socio-economic and rural–urban settings.^5^^,^^6^

**FIGURE 2:**
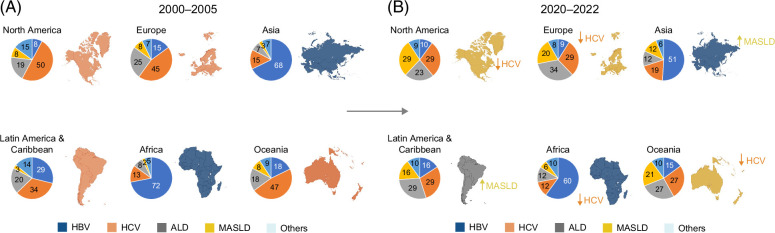
Temporal changes in the etiologic distribution of hepatocellular carcinoma (HCC) by world region. (A) 2000–2005 and (B) 2020–2022. Pie charts show the percentage contribution of hepatitis B virus (HBV), hepatitis C virus (HCV), alcohol-associated liver disease (ALD), metabolic dysfunction–associated steatotic liver disease (MASLD), and other causes within each region. HCV-related HCC declined across most regions over time, whereas MASLD-related HCC increased globally. HBV remains the leading cause in Asia and Africa.

The worldwide incidence and mortality of HCC remain highest in East Asia and Sub-Saharan Africa, where HBV is the predominant etiology, accounting for over 50% of HCC cases.^4^^,^^7^ In contrast, North America and Western Europe have seen a dramatic shift: HCV was historically the leading cause, but the proportion of HCV-related HCC has declined rapidly since 2015, with MASLD and ALD now accounting for a growing share of cases.^8^ In Eastern Europe and Central Asia, ALD is a predominant driver of cirrhosis and HCC, as these regions record some of the highest disability-adjusted life-year (DALY) rates from alcohol use worldwide, while HCV prevalence also ranks among the world’s highest.^9^ The Middle East and North Africa are characterized by a mixed profile, with HBV, HCV, and MASLD each accounting for 20%–30% of cases.^9^^,^^10^ Across Latin America, MASLD and ALD are emerging as major causes of HCC, reflecting rising obesity and alcohol consumption trends, whereas HCV-related HCC persists in countries with limited access to diagnosis and curative antiviral therapy^9^^,^^11^ (Figure 2).

## REGIONAL PERSPECTIVES IN HCC EPIDEMIOLOGY

### Africa

HCC in adult populations across Africa is distinguished by a high incidence, early age of onset, and a predominance of viral etiologies. The epidemiologic patterns differ markedly between Northern Africa, where HCV predominates, and Sub-Saharan Africa (SSA), where HBV and aflatoxin exposure are the principal drivers of HCC.^7^ Hazardous alcohol use, metabolic dysfunction, obesity, and diabetes are emerging contributors, particularly in Northern and Southern Africa, where urbanization and lifestyle changes are increasing the prevalence of MASLD.^12^ The synergistic effect of these risk factors with chronic viral hepatitis and aflatoxin exposure amplifies HCC risk.^13^ HIV co-infection further increases the HCC risk, especially in regions with high HIV prevalence, due to improved survival of HIV-infected individuals and increased risk of HBV/HCV co-infection.^12^^,^^14^

Northern Africa—especially Egypt—has historically exhibited some of the world’s highest HCC incidence rates (ASIR 13–15 per 100,000),^4^^,^^7^ mainly due to mid-20th-century parenteral antischistosomal therapy campaigns that drove widespread HCV transmission.^4^^,^^15^ Although the 2014 national HCV elimination program reduced active HCV infection by over 90%, HCC incidence has not yet declined—likely due to the large number of individuals already living with advanced fibrosis.^16^^,^^17^

Sub-Saharan Africa, encompassing Western, Eastern, Central, and Southern regions, also exhibits high HCC incidence rates (ASIR 4–10 per 100,000), with the highest rates in West and East Africa.^4^ The median age at diagnosis is 30–50 years, which is among the youngest worldwide, likely due to early-childhood HBV transmission and exposure to Aflatoxin B1, which leads to mutations in TP53, accelerating hepatocarcinogenesis.^7^^,^^12^ Indeed, a large multicenter study showed that almost 40% of HBV-related HCC in the region occurs before age 40.^18^ Hepatitis D virus (HDV) is an underrecognized but important contributor to HCC in Africa. HDV co-infection accelerates fibrosis progression and accounts for roughly 20% of HBV-associated HCC, yet diagnostic capacity and routine HDV testing remain extremely limited.^19^

Most HCC cases in Africa are diagnosed at advanced stages, with about 95% presenting as Barcelona Clinic Liver Cancer (BCLC) C or D. Survival remains among the lowest worldwide, with a median survival of 1–8 months and 3-year survival generally under 20%.^20^ The SURVCAN-3 study found a 3-year net survival of just 18.1% across 11 Sub-Saharan African countries.^21^ A systematic review showed that only 6% of patients received curative treatment, while 84% received supportive care alone.^20^ These outcomes reflect delayed diagnosis, limited surveillance, and restricted access to curative or systemic therapies. Substantial heterogeneity exists across the continent in terms of resources, thus leading to important gaps in outcomes. Indeed, countries like Egypt and South Africa have a wider capacity of resources, including liver transplant, while regions in mid-Africa lack, at times, basic resources. This was dramatically reflected in a multicenter study showing that 76% of individuals with HCC in Egypt received some form of HCC treatment, while only 3% did so in the rest of the continent.^7^ HCC in Africa has major public health implications due to its early onset and poor survival.^12^ It places a heavy socioeconomic burden by affecting adults in their most productive years and straining already limited healthcare systems.^13^

Primary prevention remains the most powerful tool for reducing HCC burden across the continent. Modeling studies suggest that achieving universal hepatitis B vaccination coverage could reduce new HCC cases by up to 50% within 3–5 decades.^22^ However, a major challenge is the substantial disparity in HBV vaccination uptake and screening between urban and rural settings, as proximity to rural areas is strongly associated with a higher burden of HBV-related HCC.^23^ These findings show that HBV vaccination and screening efforts are concentrated in urban areas, leaving rural communities with lower preventive coverage and a higher risk of chronic HBV infection and subsequent HCC. Additional measures for prevention include pre-harvest and post-harvest aflatoxin control, which has been moderately implemented in the region. Secondary prevention through targeted surveillance of high-risk populations also remains crucial.^24^ Innovative community-based strategies offer an additional pathway to overcome longstanding geographic and logistical barriers. A recent mobile HBV-HCC screening initiative in rural Tanzania demonstrated the feasibility of decentralizing surveillance to underserved communities.^25^^,^^26^ Finally, expanding access to antiviral therapy for HBV and HCV infection is a cost-effective strategy, recently supported by the new WHO guidelines, that significantly lowers long-term HCC risk, yet remains unavailable to most patients in low-resource settings.^27^ Table 1 summarizes the common and region-specific unmet needs identified across global regions to highlight priority areas for targeted intervention (Box 1).

**TABLE 1:** Common and region-specific unmet needs in hepatocellular carcinoma

Region	Commonly reported gaps	Region-specific unmet needs
Africa	• Limited surveillance • Advanced-stage diagnosis (95% BCLC C/D) • Restricted access to curative/systemic therapy • Limited antiviral access	• Rural–urban HBV vaccination disparity • Limited HDV diagnostic capacity • Incomplete aflatoxin control • Marked treatment access disparity outside Egypt and South Africa
Asia	• Incomplete surveillance uptake • Rising MASLD and alcohol-associated disease	• Persistent early-life HBV transmission • Limited cancer registry coverage in South Asia
Europe	• Low adherence to guideline-based surveillance • Socioeconomic disparities in stage and survival	• Uneven DAA access among marginalized populations • Persistent alcohol-associated HCC in Northern and Eastern Europe
North America	• Low semiannual screening adherence (~8%) • Advanced-stage presentation in disadvantaged groups	• Rural mortality is increasing faster than urban • Racial/ethnic disparities in incidence and outcomes • Limited access to specialty care in rural areas.
Latin America	• Late-stage diagnosis • Absence of organized screening programs • Limited diagnostic imaging infrastructure	• Incomplete cancer registries • Gaps in HBV/HCV screening and diagnosis • Underfunded National Cancer Control Plans
Oceania	• Late-stage presentation • Limited access to curative therapies in the Pacific Islands	• Severe resource constraints in Pacific Island nations • Persistent disparities in Indigenous and remote populations

Abbreviations: BCLC, Barcelona Clinic Liver Cancer; DAA, direct-acting antiviral; HBV, hepatitis B virus; HCV, hepatitis C virus; MASLD, metabolic dysfunction–associated steatotic liver disease.

Box 1

**Table:** Key epidemiologic changes in Africa

• HBV vaccination and treatment have expanded, but rural areas face disparities in access to care.
• MASLD and alcohol use are rising as new contributors to HCC, on top of longstanding HBV and aflatoxin exposure.
• HDV co-infection is increasingly recognized as an important driver of severe liver disease and subsequently HCC.

### Asia

HCC is a leading driver of primary liver‑cancer morbidity and mortality in Asia, accounting for 68% of global cases. East Asia alone contributes over half of all cases and deaths as of 2018.^28^ Approximately 17% of new cases occur in individuals aged 15–49 years, reflecting early-life HBV infection.^28^^–^^30^ Chronic HBV infection remains the predominant driver of HCC,^31^ with chronic HCV infection contributing in certain populations.^32^ Other factors include aflatoxin exposure, ALD, and the growing burden of MASLD.^31^^,^^32^

East Asia remains a major epicenter of HCC. The ASIR ranges from 18 to 25 per 100,000, though substantial national variation exists. For example, Mongolia reported the world’s highest ASIR at 93.7 per 100,000 and ASMR 75.4 per 100,000 in 2018, secondary to its high rates of HBV and HDV co-infection.^28^ Despite modest declines in both ASIR and ASMR over the past decade, the absolute burden continues to grow due to population aging.^29^^,^^33^ Mortality closely mirrors incidence (ASMR 17–22 per 100,000), reflecting persistently poor survival in advanced disease; however, gradual declines in China, Japan, and South Korea highlight the positive impact of HBV vaccination, antiviral therapy, and screening programs.^33^^,^^34^

**FIGURE 3:**
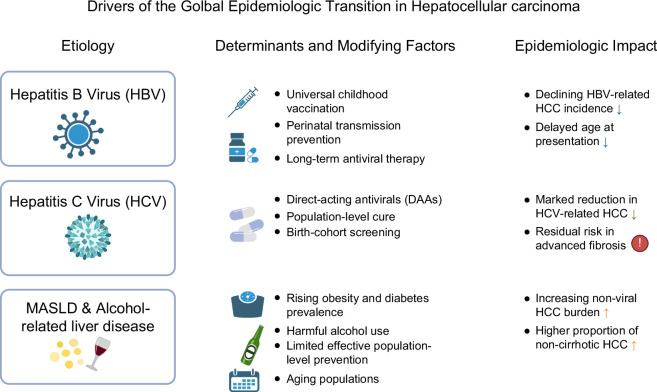
Drivers of the global epidemiologic transition in hepatocellular carcinoma. Abbreviations: HBV, hepatitis B virus; HCC, hepatocellular carcinoma; HCV, hepatitis C virus; MASLD, metabolic dysfunction–associated steatotic liver disease.

Chronic HBV has historically been the leading cause of HCC in East Asia, accounting for over 60% of cases.^8^^,^^31^ Universal HBV vaccination has substantially reduced HBV prevalence and HCC incidence—for example, in Taiwan, neonatal vaccination lowered chronic HBV from 10% to 0.5% and reduced HCC incidence and mortality by up to 80% and 63%.^35^^,^^36^ Similar trends are seen elsewhere: China’s HBV-related liver-disease mortality fell from 23.9 to 10.7 per 100,000 between 1990 and 2021.^34^^,^^37^ While viral etiologies are declining, non-viral etiologies are rising sharply. The prevalence of MASLD in Asia has reached ~34%, and MASLD-related HCC now represents the fastest-growing cause of liver cancer in the region.^32^^,^^37^ Rising alcohol consumption in China, Korea, and Japan, and the resulting increase in ALD, can act synergistically with viral hepatitis to raise HCC risk by as much as 5-fold.^27^ Although aflatoxin exposure continues to play a role in certain rural regions of China, its impact is diminishing with improved food safety regulations.^35^^,^^38^ Key drivers of the global epidemiologic transition in HCC are shown in Figure 3.

South-Eastern Asia also remains a high-incidence region for HCC, with ASMR typically 10–18 per 100,000.^28^^,^^29^ HBV infection remains the dominant etiology, responsible for 55%–70% of HCC cases.^8^^,^^33^ HCV infection contributes a smaller but significant proportion, particularly in Thailand and Vietnam, where unsafe medical injections and blood transfusions historically facilitated transmission.^38^

In South Asia, India and Pakistan represent the largest share owing to their large populations and the high prevalence of major risk factors, particularly HBV and HCV infections. The ASIR for liver cancer approximates 2.4 per 100,000 in India and 4.0 per 100,000 in Pakistan. Other countries in the region, including Bangladesh, Sri Lanka, Nepal, Bhutan, Maldives, and Afghanistan, report lower absolute case numbers but exhibit high MIRs, frequently exceeding 0.85.^29^^,^^39^^,^^40^ The accuracy of HCC incidence and mortality estimates in South Asia is limited by substantial gaps in cancer registry, with the true burden likely underestimated^41^^,^^42^ (Box 2).

Box 2

**Table:** Key epidemiologic changes in Asia

• HBV vaccination and antiviral therapy, as well as widespread HCV DAA treatment, have reduced viral hepatitis–related HCC in younger generations.
• MASLD and alcohol use are growing rapidly and driving more HCC cases across East, Southeast, and South Asia.
• Early-onset and non-cirrhotic HBV-related HCC are now better recognized, prompting renewed focus on risk stratification and screening.

### Europe

The ASIR for HCC is ~6–7 per 100,000, and ASMR of 5–7 per 100,000.^1^ Geographic disparities persist, with the highest incidence and mortality in Southern and Eastern Europe, reflecting historical burdens of viral hepatitis and ALD, as well as the growing contribution of MASLD.^43^ HBV is more prevalent in Eastern and Southern Europe, while HCV is a major driver in Southern Europe, particularly Italy, Spain, and Greece. ALD is most prominent in Northern and Eastern Europe, and MASLD, obesity, and diabetes are increasingly important in Western and Northern Europe.^44^

**FIGURE 4:**
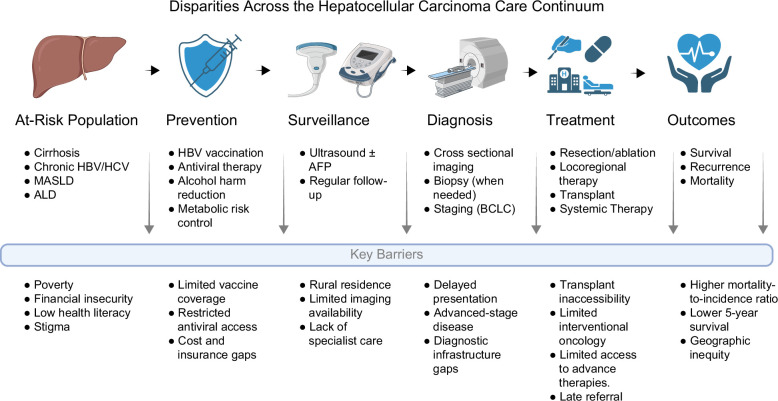
Disparities across the hepatocellular carcinoma (HCC) care continuum. Abbreviations: ALD, alcohol-associated liver disease; BCLC, Barcelona Clinic Liver Cancer; HBV, hepatitis B virus; HCC, hepatocellular carcinoma; HCV, hepatitis C virus; MASLD, metabolic dysfunction–associated steatotic liver disease.

Adherence to HCC surveillance in the region remains poor, despite society recommendations, as evidenced by a 2025 meta-analysis of over 1.2 million patients in which only 54% underwent any surveillance, and just 8.8% received guideline-concordant biannual screening.^45^ Socioeconomic disparities exert a profound influence on HCC incidence and outcomes. Individuals with lower income, limited education, migration background, or residence in deprived neighborhoods have higher incidence rates, later-stage presentation, and worse survival^46^ (Figure 4).

Public health interventions have yielded measurable, though regionally variable, success. Universal HBV vaccination has significantly reduced HBV prevalence and HCC incidence in vaccinated cohorts, with over 90% coverage achieved in approximately three-quarters of European countries with infant vaccination programs.^47^ The implementation of DAAs for HCV has been transformative: in Italy, liver-related mortality is projected to decline by 75% by 2030.^48^ However, progress remains uneven due to limited DAA access and underdiagnosis among marginalized populations.^48^^,^^49^ Conversely, alcohol-associated HCC continues to rise across Northern and Eastern Europe, following increasing per capita consumption and persistent policy gaps.^16^^,^^27^^,^^45^^,^^49^^,^^50^ Sustained efforts are needed to expand surveillance coverage, address the rising burden of metabolic risk factors, and reduce socioeconomic and geographic disparities in access to care (Box 3).

Box 3

**Table:** Key epidemiologic changes in Europe

• HCV-related HCC has fallen sharply due to universal access to DAAs.
• MASLD-related HCC is rising rapidly in Western and Northern Europe, paralleling increasing obesity and diabetes prevalence.
• Alcohol-associated HCC predominates in Northern and Eastern Europe, reflecting higher per capita alcohol consumption and persistent policy gaps.

### North America

In the United States, the ASIR rose steadily from the 1970s to a peak of 10.03 per 100,000 in 2015, then gradually declined to 9.20 per 100,000 by 2019, according to the Surveillance, Epidemiology, and End Results (SEER) Program.^51^ Mortality trends have closely mirrored incidence. Notably, liver cancer mortality has increased faster than any other major cancer site in recent decades, reflecting both rising incidence and persistently poor survival.^52^ However, projections indicate a potential rise of HCC by 2040, as ALD and MASLD increasingly replace viral hepatitis as the dominant etiologies.^51^^,^^53^^,^^54^ In Canada, the 5-year survival is poor at 19%, underscoring the high case-fatality of this malignancy in the region.^55^^–^^57^ Within North America, substantial geographic heterogeneity exists: in the United States, the highest rates are observed in the West and South.^58^ Whereas in Canada, higher rates cluster in provinces with larger immigrant populations from HBV/HCV-endemic regions and in socioeconomically deprived areas.^55^^–^^58^

The etiologic profile of HCC in North America has shifted markedly. HCV, HBV, and ALD once predominated, with HCV accounting for ~20%–22% of cases and HBV 4%–6% in 2014.^59^^–^^61^ Following widespread DAA adoption and improved HBV vaccination and antiviral therapy, HCC incidence has plateaued and begun to decline.^8^^,^^53^^,^^61^ Meanwhile, MASLD and ALD have become the major contributors to HCC, with MASLD now responsible for 23%–36% of U.S. cases and representing the fastest-growing etiology, especially among Hispanic, White, and Asian populations.^8^^,^^62^

Marked demographic and socioeconomic disparities define the epidemiology of HCC in North America. Racial and ethnic minorities, including Hispanic, Black, Asian/Pacific Islander, and American Indian/Alaska Native populations, experience substantially higher incidence and mortality compared with non-Hispanic Whites.^51^ These disparities reflect varied exposure to risk factors as well as healthcare access, insurance coverage, and timely receipt of curative therapy.^8^^,^^62^^–^^66^ Prior data have shown that patients with public insurance or no insurance, lower income, or residence in rural and high-poverty neighborhoods are more likely to present with advanced-stage disease, less likely to receive curative therapy, and experience higher mortality.^62^^–^^67^

North America faces similar challenges to Europe implementation of guideline-directed surveillance for HCC. Surveillance utilization consistently falls below 50% among at-risk populations, and only around 8% of eligible individuals undergo the recommended semiannual screening intervals^45^ (Box 4).

Box 4

**Table:** Key epidemiologic changes in North America

• HCC incidence has begun to plateau or decline as HCV-related cases decrease in the DAA era.
• MASLD and alcohol use are now the fastest-growing causes of HCC.
• HCC incidence and mortality are highest among Hispanic, Black, and Asian/Pacific Islander populations, reflecting persistent racial, ethnic, and socioeconomic disparities.

### Latin America

Latin America represents an intermediate-burden region for HCC, characterized by substantial regional heterogeneity, evolving etiologic patterns, and persistent structural barriers that delay diagnosis and limit access to curative therapies.^68^ Across the region, ASIRs generally between 3.5 and 5.5 per 100,000.^1^^,^^67^ Incidence varies widely, the Caribbean and Central America report the highest rates (approaching 5.5 per 100,000), the Southern Cone (Argentina, Chile, Uruguay, southern Brazil) the lowest (3.5–4.5 per 100,000), and the Andean region and parts of Central America intermediate rates, mirroring variation in viral hepatitis burden and other risk factors.^67^

Over the past 2 decades, HCC incidence has risen steadily across Latin America. In Brazil, hospitalization rates increased from 2.1 to 5.8 per 100,000 between 2005 and 2018.^69^^,^^70^ The region’s etiologic profile reflects both a historic burden of viral hepatitis and an ongoing shift toward metabolic and ALD, resulting in substantial subregional heterogeneity. In South America, a multicenter study showed that HCV accounted for 48% of HCC cases, followed by ALD (22%), HBV (14%), and MASLD (9%) in 2015.^6^ Interestingly, a follow-up study by the same group 8 years later showed a marked increase in MASLD-related HCC.^71^ The Andean region and parts of Central America continue to face a high burden of HBV-related HCC, with HBV responsible for up to 45% of liver cancer deaths, compared with only 6% in the Southern region.^72^ HBV, however, has been found to be a major cause of early-onset HCC in the region.^73^ Alcohol is a major contributor throughout Latin America and exceeds HBV or HCV as a cause for HCC mortality in several Southern countries.^8^^,^^72^ As rates of obesity, diabetes, and metabolic syndrome climb rapidly, MASLD is emerging as an increasingly important etiology, particularly in urbanized and economically developed areas.^8^^,^^62^^,^^74^

HCC in Latin America is frequently diagnosed at advanced stages, leading to poor survival. In a multicenter cohort from 6 South American countries, only 47% of cases were detected through surveillance, and lack of surveillance was strongly linked to higher mortality.^6^ Late-stage presentation reflects multiple barriers, including the absence of organized screening programs, under-resourced primary care, limited diagnostic imaging, incomplete cancer registries, and especially pronounced gaps in rural areas.^75^^,^^76^ Socioeconomic inequality contributes to the rising incidence observed in Central America, the Caribbean, and rural Brazil, where delayed diagnosis and limited treatment availability remain the prevailing pattern.^76^

Across Latin America, multiple public health initiatives have been implemented with variable impact. Universal newborn HBV vaccination, recommended by the Latin American and Caribbean Code Against Cancer, is widely adopted and has reduced HBV-related HCC where coverage is high.^77^ However, gaps in HBV/HCV screening and diagnosis remain substantial.^8^ National Cancer Control Plans exist in only 16 countries, are often underfunded, and frequently lack robust cancer registries, limiting coordinated implementation^77^ (Box 5).

Box 5

**Table:** Key epidemiologic changes in Latin America

• HCC incidence continues to rise, especially outside major cities where access to care is limited.
• MASLD and alcohol are replacing HCV as leading causes of HCC in many Latin American countries.
• HBV vaccination has reduced infection in younger cohorts, but gaps in viral hepatitis testing and treatment persist.

### Oceania

The epidemiology of HCC in Oceania is marked by substantial heterogeneity. According to GLOBOCAN 2022, Australia reported an ASIR of 4.2 per 100,000 in 2022, while New Zealand reported an ASIR of 3.7 per 100,000, figures that have steadily increased over the past 2 decades.^1^ The rising rates follow the increasing population-level prevalence of obesity, diabetes, metabolic syndrome, and MASLD, despite declining viral hepatitis–related disease in the DAA era.^32^^,^^78^ In contrast, several Pacific Island nations experience disproportionately high HCC burdens: Tonga reports an ASMR near 8.5 per 100,000, among the highest globally, while Micronesia, the Marshall Islands, and Papua New Guinea report ASMRs ranging from 7.2 to 10.2 per 100,000.^1^^,^^29^^,^^32^^,^^40^^,^^79^

Survival outcomes for adults with HCC in Oceania remain poor, though modest improvements have been observed in Australia and New Zealand. In Australia, 5-year survival rates are ~21%, with median survival ranging from 12 to 18 months.^80^^–^^82^ New Zealand shows similar outcomes.^80^^,^^83^^,^^84^ In the Pacific Islands, survival is below 20% with median survival around 12 months, reflecting late-stage presentation and extremely limited access to curative therapies.^84^^–^^86^ Although early-stage diagnoses and access to curative therapy have improved over time, remote, Indigenous, and socioeconomically disadvantaged populations face persistent disparities in early detection and treatment access, and survival^87^^,^^88^ (Box 6).

Box 6

**Table:** Key epidemiologic changes in Oceania

• HCC incidence is rising in Australia and New Zealand due to increasing metabolic risk factors, despite declines in viral hepatitis–related disease.
• Pacific Island nations continue to experience some of the world’s highest HCC mortality rates, driven by chronic HBV infection and rapidly escalating obesity and diabetes.
• Late-stage diagnosis, especially in Indigenous and remote communities, contributes to poor survival and persistent disparities across the region.

### Rural–urban disparities in global HCC burden

Rural–urban disparities in HCC reflect substantial global variation in risk factors, healthcare access, and social determinants of health. The world’s highest HCC incidence and mortality occur in regions with large rural populations, including East and Southeast Asia, SSA, and parts of Central America.^1^^,^^9^^,^^24^^,^^28^ In these settings, limited access to HBV vaccination, antiviral therapy, screening infrastructure, and diagnostic imaging contributes to persistently elevated ASIR and MIR.^1^^,^^4^ Rural communities additionally face disproportionate food insecurity and inadequate regulation of crop storage in certain parts of the world, sustaining high aflatoxin B1 exposure that synergistically increases HCC risk.^9^^,^^24^ In addition, rural residents experience delayed diagnosis, and consequently are at higher risk for advanced-stage disease, and substantially reduced eligibility for curative therapy.^4^^,^^24^^,^^74^^,^^89^

In high-income countries, such rural populations have also experienced a markedly faster rise in both incidence and mortality over the past 2 decades. In the United States, HCC mortality increased nearly twice as fast in rural areas compared with urban areas between 2005 and 2023, with rural mortality surpassing urban rates by 2020.^23^^,^^90^ These trends reflect higher rural prevalence of obesity, diabetes, MASLD, and chronic alcohol use, compounded by limited access to specialty care and lower adherence to surveillance.^8^^,^^24^^,^^40^^,^^91^^,^^92^ Rural patients are consistently less likely to undergo semiannual surveillance, and less likely to receive ablation, or transplantation, even after adjustment for race, income, and insurance status.^23^^,^^40^^,^^91^^,^^92^

### Epidemiology of non-cirrhotic HCC

Non-cirrhotic HCC represents a clinically significant subset of primary liver cancers whose global burden is steadily increasing. Contemporary multicenter studies indicate that 10%–22% of all HCC cases occur in non-cirrhotic livers.^93^^–^^97^ This proportion varies by region, reflecting local prevalence of metabolic disease, viral hepatitis, and environmental carcinogens. In the United States, non-cirrhotic HCC accounts for ~10%–12% of cases, while studies from the Netherlands and broader Europe report proportions up to 22%, with a 61% increase in non-cirrhotic HCC incidence over a decade.^94^^,^^95^ In East Asia, where chronic HBV is highly endemic, 25.7% of HCC occurs in non-cirrhotic individuals, with HBV as the leading cause.^96^

MASLD is now the fastest-growing etiology of non-cirrhotic HCC worldwide, reflecting the parallel rise in obesity, diabetes, and metabolic syndrome.^16^^,^^27^^,^^98^ Although the annual incidence of MASLD-related HCC among non-cirrhotic individuals is relatively low (0.1–1.3 per 1000 patient-years), the enormous size of the affected population translates into a substantial absolute burden.^98^ In parallel, aflatoxin B₁ remains a potent driver of non-cirrhotic HCC in parts of Sub-Saharan Africa and Asia.^5^^,^^99^^,^^100^ Accurate quantification of cirrhotic/non-cirrhotic shift remains constrained by inconsistent assessment of liver fibrosis at the time of HCC diagnosis. As metabolic risk factors continue to expand worldwide, particularly in high-income countries and rapidly urbanizing regions, non-cirrhotic HCC is poised to become an increasingly significant contributor to the global liver cancer burden.

## CONCLUSIONS

HCC remains a major global health challenge, marked by profound geographic heterogeneity, shifting etiologic patterns, and persistent inequities in prevention and care. In many parts of Asia and Africa, viral hepatitis continues to dominate, compounded by aflatoxin exposure, HDV co-infection, and limited access to screening and curative therapy. In contrast, North America, Europe, and increasingly Latin America are witnessing a transition toward MASLD-driven and ALD-driven HCC, reflecting global epidemics of obesity, diabetes, and harmful alcohol use.

Future progress will require coordinated global strategies that address the full spectrum of disease determinants with sustained investment. Scaling up HBV vaccination, including birth-dose administration, expanding access to HBV and HCV antiviral therapy, and strengthening food safety to reduce aflatoxin exposure are critical in high-incidence regions. Simultaneously, comprehensive public health initiatives targeting metabolic disease, obesity, and alcohol use are essential to curb the rising wave of MASLD-related and ALD-related HCC. Aligning regional strategies with global health policies will likely offer the most promising path toward reducing the rising burden of HCC worldwide.
